# One-Year Demographical and Clinical Indices of Patients with Chronic Disorders of Consciousness

**DOI:** 10.3390/brainsci11050651

**Published:** 2021-05-16

**Authors:** Julia Nekrasova, Mikhail Kanarskii, Ilya Borisov, Pranil Pradhan, Denis Shunenkov, Alexey Vorobiev, Maria Smirnova, Vera Pasko, Marina V. Petrova, Elena Luginina, Igor Pryanikov

**Affiliations:** 1 Department for the Study of Chronic Disorder of Consciousness, Federal Research and Clinical Center of Intensive Care Medicine and Rehabilitology, 107031 Moscow, Russia; ynekrasova@fnkcrr.ru (J.N.); kanarmm@yandex.ru (M.K.); realzel@gmail.com (I.B.); dshunenkov@fnkcrr.ru (D.S.); avorobiev@fnkcrr.ru (A.V.); ipryanikov@fnkcrr.ru (I.P.); msmirnova@fnkcrr.ru (M.S.); vpasko@fnkcrr.ru (V.P.); mail@petrovamv.ru (M.V.P.); eluginina@fnkcrr.ru (E.L.); 2 Department of Anestesiology-Reanimatology, People’s Friendship University of Russia, 117198 Moscow, Russia

**Keywords:** chronic disorder of consciousness, vegetative state, survival rate, recovery of consciousness, prognosis

## Abstract

This work aims to evaluate the prognostic value of the demographical and clinical data on long-term outcomes (up to 12 months) in patients with severe acquired brain injury with vegetative state/unresponsive wakefulness syndrome (VS/UWS/UWS) or a minimally conscious state (MCS). Patients (*n* = 211) with VS/UWS/UWS (*n* = 123) and MCS (*n* = 88) were admitted to the Federal Research and Clinical Center of Intensive Care Medicine and Rehabilitology after anoxic brain injury (*n* = 53), vascular lesions (*n* = 59), traumatic brain injury (*n* = 93), and other causes (*n* = 6). At the beginning of the 12-month study, younger age and a higher score by the Coma Recovery Scale-Revised (CRS-R) predicted a survival. However, no reliable markers of significant positive dynamics of consciousness were found. Based on the etiology, anoxic brain injury has the most unfavorable prognosis. For patients with vascular lesions, the first three months after injury have the most important prognostic value. No correlations were found between survival, increased consciousness, and gender. The demographic and clinical characteristics of patients with chronic DOC can be used to predict long-term mortality in patients with chronic disorders of consciousness. Further research should be devoted to finding reliable predictors of recovery of consciousness.

## 1. Introduction

Severe traumatic brain injuries (TBI), vascular lesions (VL), respiratory or cardiac arrest, and gross metabolic impairments often lead, in the outcome of a coma, to the formation of disorders of consciousness (DOC) such as vegetative state/unresponsive wakefulness syndrome (VS/UWS) or minimally conscious state (MCS) [1,2]. According to the duration, VS/UWS is divided into transient (less than 1 month) and chronic or persistent (up to 3 months in the non-traumatic cases and up to 12 months in traumas). Above the indicated periods, it is customary to speak of permanent VS/UWS with prognostically minimal chances of regaining consciousness [3,4,5]; however, according to the latest recommendations [6], the very term “permanent” in relation to VS/UWS is not advised. MSC chronization is usually not emphasized [6]. Persistent DOC develops in 1–14% of TBI patients, and around 12% in non-traumatic injuries [7].

In connection with the significant increase in the number of such patients all over the world, studies of the evolution of this condition and the search for methods to identify its prognosis are of particular relevance. Most often, the search for such methods is carried out in the field of instrumental diagnostics and electrophysiology; however, a comparison of demographic, clinical, and follow-up data can also help identify prognostic markers of survival and outcome of the disease, determine an adequate vector of the rehabilitation route, personify cognitive rehabilitation, and correctly inform the patient’s relatives and social services.

A number of publications are devoted to this issue, the main ones of which are shown in Table 1.

The pioneer works of Nakase-Richardson et al. [8] and Higashi et al. [9] at the end of the 20th century showed low survival rates and a small percentage of consciousness improvement in DOC patients. Luaute et al. (2010) [10] and Estraneo et al. (2010) [11] initiated modern major studies for these groups of patients. Thus, Luaute et al. [10] revealed that patients with a higher level of consciousness (MCS+) demonstrate a higher survival rate and rehabilitation potential. In their work, Baricich et al. [14] observed patients in VS/UWS for four years showing a 40% survival rate, which is comparable to the data obtained in the study by Aidinoff et al. [15]. However, it is strikingly different from the results demonstrated in the work of Pascarella et al. [18], where the six-year survival rate hovers around 70%, probably due to the large proportion of patients in MCS. In recent papers of Estraneo et al. (2019, 2020) [19,21] the level of consciousness is not a predictive marker of survival for long-term (up to 3 years) studies. At the same time, an increase in the level of consciousness both for patients in the VS/UWS and for patients in the MCS occurs in the first year (or even in the first six months) of the study. This makes the abovementioned timeframe an important factor in early rehabilitation and, therefore, the most interesting period in terms of assessing the rehabilitation potential of patients.

Several studies identified a number of predictors of favorable clinical outcome in DOC patients, such as younger age, shorter time after brain injury, and traumatic etiology [12,13,22,23]. In addition, the female gender combined with higher CRS-R scores was also identified as a predictor of recovery of consciousness during the first 12 months after the injury [11]. However, it seems that reliable predictors of the outcome of DOC have not yet been found.

Moreover, some of the studies do not provide information about the level of consciousness to which patients performance improved. In some cases, this is due to the complexity of assessing the level of consciousness outside the hospital, yet even in a hospital setting, differential diagnostics between VS/UWS and MCS−, as well as between MCS+ and consciousness, is not always reliable. For example, mirror tracking, which is used to interpret the level of DOC, is not always an accurate method of differentiating between VS/UWS and MCS− [24]. The presence of a tetraplegia, which is often found in this group of patients, does not allow for the localization of pain [25]. This increases the number of diagnostic errors, resulting in statistical biases. In this regard, the most objective approach for statistical analysis seems to be based on the reduction of possible levels of consciousness to two functional units associated with the presence or absence of communication (contact with the patient).

In this study, we analyzed the mortality rate and changes in the level of consciousness for DOC patients based on demographic and clinical data, such as the etiology, age, gender, and the CRS-R scores, to identify significant prognostic indices for the disease course.

## 2. Materials and Methods

### 2.1. Participants

This longitudinal cohort study involved 211 patients (122 men and 89 women) with chronic DOC, who were admitted to Federal Research and Clinical Center of Intensive Care Medicine and Rehabilitology from 2016 to October 2019.

Patients with left-hemisphere ischemic strokes were excluded from the study. The issue of total aphasia, agnosia, and apraxia, and differential diagnosis of MCS− from MCS+ in patients with these conditions, is currently an unsolved problem; therefore, to achieve statistical integrity of the study, this group of patients was not considered. CT and/or MRI scans were made to exclude brainstem lesions and confirm the diagnosis of VS/UWS and MCS. Exclusion criteria also involved previous TBI, mental, or neurodegenerative diseases in medical history, as well as age over 80 years.

The common etiologies comprised TBI, anoxic brain injury (ABI), and vascular lesions. Rare cases were registered due to infection of the central nervous system and neurodegenerative diseases.

### 2.2. Assessment of the Level of Consciousness and Diagnostic Procedure

At study entry, two skilled neurologists assessed the patients independently and at different times to confirm the diagnosis of DOC in accordance with standardized clinical criteria for VS/UWS, MСS–, and MСS+ by means of the Russian version of the CRS-R scale. During assessment, all patients were compensated for electrolyte, metabolic, infectious, and other disorders that could lead to depression of consciousness, as well as affect the level of awareness. The CRS-R score was checked weekly during the hospital stay. At every assessment, medical personnel treating the patient were thoroughly interviewed in order to take into account fluctuations in the level of consciousness. If personnel noted any changes concerning communication, gaze fixation, or localizing pain signals, additional assessments were performed more frequently.

In cases where neurologists disagreed with the diagnosis, instrumental diagnostic methods, such as EEG, PSG, MRI, functional MRI, PET, and international recommendations and publications in authoritative sources were used to expose a single form of DOC. All patients received neuroprotective and metabolic therapies along with physiotherapy, massage, individual rehabilitation programs, therapeutic physical training, speech therapy, and cognitive behavioral therapy with a psychologist.

### 2.3. Follow-Up Data Collection

After patients were discharged, their condition was monitored by direct contact with the relatives or caregivers during the first 12 months.

The key issue in contact with the patients’ relatives was the determination of the level of consciousness, taking into account all possible limitations when they were exposed remotely. We proposed a method of terminology reduction, with the help of which we focused on two fundamentally opposite levels of cognition: absence and presence of communication. The lack of communication or dubious communication by all available methods, bearing in mind the level of consciousness at discharge, made it possible to include patients that were presumably in VS/UWS and MCS− in the category of “non-communicating” participants. Presence of communication consolidated patients in MCS+, emerging from MCS, and conscious state.

If the relatives had doubts about the presence or absence of communication, as well as when the relatives recorded single manifestations of awareness, the patient belonged to the group of non-communicating participants. In cases where relatives or caregivers of the patient noted that the patient communicates with them using eyes or gestures and the previous level of consciousness established in the hospital was VS/UWS, MCS−, or MCS+, the conferred level of consciousness was MCS+ or “emergence from MCS”. If the patient, being at home, began to talk, confidently communicate not only by signs, but also verbally, the patient was assigned the status of “conscious”. It is easy to see that such a separation between MCS+ and consciousness is not reliable enough, as a result of which it was decided to combine them into one group of communicating patients.

It should be noted, however, that the optimal method for detecting cognition in a remote mode is videoconference with the ability to directly see the patient when determining the level of consciousness. For a number of technical reasons, this method was not available for all patients at the time of the study and, as a result, was not used in the work.

In the event of the patient’s death, the date of death and the level of consciousness before death (communicates/does not communicate) were recorded.

### 2.4. Statistical Data Analysis

Data were analyzed using the program STATISTICA 10 (StatSoft.Inc).

Quantitative data were presented as a mean and a standard deviation. Nominal values were described with absolute values and percentages. The Kolmogorov-Smirnov test was used to assess the nature of the distribution of interval variables. We used non-parametric Mann–Whitney U-test for continuous variables and Chi-square for categorical variables. A *p*-value *<* 0.05 was considered statistically significant.

To investigate the relationships between predictors at baseline and outcome at 12 months post-injury, we applied two regression models. In the first model, we included all 211 patients and investigated selected variables like gender, age, etiology, CRS-R scores, and the level of consciousness as independent predictors of the outcome (dependent variable). In the second model we analyzed survived patients (*n* = 137) and included selected variables as independent predictors and the outcome as the dependent variable.

### 2.5. Legal Issues

Everyone acquired informed consent explaining the details of the study. With patients being unable to provide his/her consent on the study, the consent form was certified by three members of the medical team presently employed at the Federal State Budgetary Institution of the Russian Federation Federal Research and Clinical Center of Intensive Care Medicine and Rehabilitology. The ethics committee approved the study on 12 May 2019 under protocol no. 08/19/22.

The research was carried out under the Helsinki Declaration adopted at the 18th General Assembly of the World Medical Association (WMA) (Helsinki, Finland, June 1964), 59th WMA General Assembly, Seoul, Republic of Korea, October 2008; article 17 of the Principles of Public Health Legislation, orders, and instructions issued by Ministry of the health of the Russian Federation.

## 3. Results

### 3.1. Demographical and Clinical Data

The demographical and clinical data of the patients participating in the study are summarized in Table 2.

In a group of 211 people, TBI predominated by etiology (44%). The greater number of males among patients with TBI is probably explained by the more active participation of this gender group in traumatic activities [26]. The second place in terms of the frequency of causes leading to DOC was occupied by vascular lesions (27.9%). The consequences of anoxic brain damage accounted for 25.1%, and other causes such as CNS infection (four patients) and brain tumor (two patients) for 2.8% of the total number of patients.

According to the level of consciousness, 58.3% were patients in the VS/UWS, 26% in MCS− and 15.7% in MCS+.

### 3.2. Mortality within First Year after Brain Injury

Patient mortality data were obtained on the 3rd, 6th, and 12th month after the event that caused DOC by collecting information from the patient’s medical history or interviewing relatives at the post-hospital stage. The choice of control points is justified by closer attention to the first year of recovery from brain damage.

According to the data obtained, within a year after the event, mortality amounted to 35% of the total number of study participants.

Out of the total number of deaths in a year, 20.3% died in the first three months. Among them, most of the patients were in VS/UWS (86.8%), with 6.6% in MСS− and the same number in MСS+ (see Table 3).

In the next 3-month long period (at 6 months after the injury), 29.7% of the total number of deaths occurred: 36.4% were in VS/UWS, 27.3% in MCS−, and 18.2% in MCS+. By this checkpoint, three patients somatically worsened, lapsed into coma, and died, and one patient improved up to the conscious state and died because of the comorbidity.

In the period from the second to the third checkpoint, 50% of the total number of deaths occurred. In the group of non-communicating patients, consisting of 178 people, 17.4% of patients died; 18.1% died in the communicating group. Thus, in the considered group, the survival rate of patients did not correlate with the level of consciousness.

### 3.3. Changes in the Level of Consciousness during the First Year after the Brain Injury

On a 3-month interval, a rather small number of patients acquired a communicative status—13.7%, and the overwhelming majority of them were initially in MCS−. Only seven patients in VS/UWS began to communicate within 3 months. The majority (74.4%) of the study participants did not change the level of consciousness (Table 4).

A similar picture was observed at the next checkpoint (6 months after the injury) (Table 5). It should be noted that there were a few significant improvements in consciousness up to the level of communication: three patients increased the level of consciousness from VS/UWS to MCS+ and 10 from MCS− to MCS + and consciousness.

Decreased number of patients who improved the level of consciousness is observed at the third checkpoint (12 months after the event) (Table 6). This indicates the fact that restoration of consciousness is most likely at an early stage of rehabilitation (up to 6 months), which makes this period a key target for rehabilitation measures.

**Figure 1:**
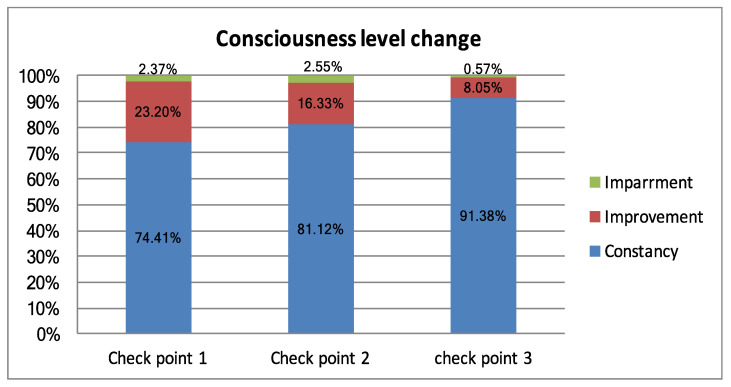
Histogram representation of changes in the level of consciousness at three control points.

Thus, during the first 3 months, every fourth patient increased the level of consciousness, whereas after 6 months, only every 10th patient achieved improvement (Figure 1).

### 3.4. Predictors of Survival and Recovery of Consciousness in Patients with Chronic DOC

The following parameters were considered as possible predictors of the outcome of the DOC:

1. The initial CRS-R score;
2. Etiology;
3. Gender and age of patients.

#### 3.4.1. The Initial CRS-R Score

We calculated and compared the mean CRS-R score of all the participants with catamnesis data to predict the survivability of patients with DOC. The mean initial CRS-R score in patients who survived the year was 8.13 ± 3.7, which is higher than in patients who died in the first 12 months after the event (6.31 ± 3.4). Thus, better 12-month prognosis in relation to survival was associated with higher CRS-R total (Me = 7 [Q1 = 5, Q3 = 10] vs. Me = 5 [Q1 = 4, Q3 = 7]) (U = 2959; *p* < 0.001). For the improvement, however, no statistical significance was found (U = 1624; *p* = 0.64).

#### 3.4.2. Etiology: Mortality and Changes in Consciousness Level

Based on the reduced classification of patients into communicating and non-communicating groups at three checkpoints (see Table 7), it can be concluded that ABI leads to chronic DOC much more often than other etiologies. It is also important to note that a few non-communicating post-anoxic patients (approximately 7.7%) started to communicate. The latter indicator significantly distinguishes the anoxic group from patients with TBI, among whom 18.8% were able to communicate within 1 year of the injury. However, 33.3% of TBI patients did not raise the level of consciousness within a year. Univariate analyses showed that in general, improvement in patients had no significant association with traumatic etiology ($\chi_{\left(1,137\right)}^{2}=0.128$, *p* = 0.72) compared to non-traumatic (ABI, VL, and others).

In a group of patients with VL, a significant increase in the level of consciousness mainly occurs in the first 3 months after the injury. In general, 23.5% of patients with DOC of this etiology increased their level of consciousness to the communicating status.

The mortality rate of DOC patients with ABI was 45.2%, the highest when compared with other etiologies. The mortality of non-communicating anoxic brain injury patients was more than 98%.

The 12-month prognosis for survival was better in traumatic patients ($\chi_{\left(1,211\right)}^{2}=9.5$, *p* = 0.002). The mortality rate of patients with DOC of traumatic etiology was 23.6%, which is two times lower than in the group with post-anoxic patients. The share of communicating patients in this group was 25.8%, out of which two patients died. Out of the non-communicating patients with TBI, 20 participants died within a year. In TBI patients, the mortality rate among non-communicating participants exceeded the mortality rate among communicating participants, which allows us to conclude that the severity of TBI is an unfavorable prognostic factor for the one-year survival rate of patients.

In the group of patients with VL, the mortality rate in the first year was 40.7%, which brings this group closer to the group of post-anoxic patients.

#### 3.4.3. Gender and Age

Univariate analyses showed that surviving patients had significantly younger age (U = 2889; *p* < 0.001) than patients who did not survive (Me = 38 [Q_1_ = 29, Q_3_ = 53] vs. Me = 57.5 [Q_1_ = 42, Q_3_ = 65]). However, improvement of consciousness had no significant association with age (U = 1603; *p* = 0.57).

Gender had no significance ($\chi_{\left(1,211\right)}^{2}=0.27$, *p* = 0.602) for the one-year survival of patients and for the improvement ($\chi_{\left(1,137\right)}^{2}=0.04$, *p* = 0.83). According to the etiology, in female patients with TBI a slightly more favorable prognosis was revealed compared to male patients in terms of mortality (17.8% versus 26.1%), as well as in relation to the improvement of consciousness (60.8% versus 50%). However, in general, it is not possible to make a reliable conclusion about the prognostic significance of gender in the sample under consideration.

#### 3.4.4. Logistic Regression Analysis

We considered the following parameters to construct a logistic regression model: gender: 0—Male, 1—Female; etiology: 0—trauma, 1—non-traumatic lesion; consciousness: 0—non-communicating patient, 1—communicating patient; outcome: 0—died, 1—survived.

Regression analysis provided a significant final model for 211 patients (likelihood ratio: χ^2^ = 46.591, *p* < 0.001). In this model younger age, and higher CRS-R total score were significantly associated with survival rate, whereas gender, etiology, and initial level of consciousness (communicating–non-communicating group) were not (see Table 8).

Then we analyzed surviving patients (*n* = 137) concerning the improvement of the level of consciousness (see Table 9).

In this analysis, 0 means no improvement, and 1 means improvement from the non-communicating to communicating group. Based on the results of univariate analyses where all variables were non-significant, it was expected that regression analysis provided an insignificant final model (likelihood ratio: χ^2^ = 5.63, *p* = 0.22). In this model age, CRS-R total score, gender, and etiology were not significantly associated with improvement for those who survived.

## 4. Discussion

Epidemiological data obtained as a result of this study indicate the prevalence of TBI among all etiologies leading to DOC, which is consistent with the data of other large studies [15]. TBI patients are on average younger than representatives of other nosological forms, which is also confirmed by the results of studies by other authors [11,13]. Thus, the sample under consideration is representative in terms of demographic data.

In the sample of 211 patients, the main predictors of survival are younger age and higher CRS-R score (initial level of consciousness). Yet, neither age no gender or any other characteristic in our study do not affect the dynamics of the level of consciousness. It should be said that in some other studies, a higher score on the CRS-R scale, revealed during the initial examination, correlates with a positive dynamics of the level of consciousness [27,28]. Our methodology, based on the division of patients by the ability communicate into two groups, did not take into account the improvements inside the communicating and non-communicating groups. For instance, initially communicating patients with MCS+ cannot have a binary outcome within the framework of the analysis (the category “improved” is not applicable to them, since we understand the improvement as a shift from the non-communicating to communicating group). It can, on the first glance, limit the study, but the low frequency of transition of non-communicating patients to the group of communicating patients casts doubt on the need for divergence of VS/UWS and MCS− in the group of non-communicating patients for long-term prognosis. Thus, we can conclude that higher CRS-R score and the level of consciousness between patients in VS/UWS and MCS− do not predict a considerably better outcome, and we cannot say that patients in MCS− are more likely to regain consciousness than patients in VS.

In terms of the etiology, the results obtained do not differ from the data of other studies [13], that is, ABI is the most unfavorable cause of DOC in terms of positive dynamics of cognition, as well as one-year survival. According to the results of the study, it can be concluded that in the group of patients with TBI, the initial level of consciousness can be considered a reliable predictor of survival. In patients with VL, we observe a high mortality comparable to the mortality of ABI patients. However, the proportion of VL patients who improved the level of consciousness after one year of observation is significantly higher. It should be noted that for this etiological group, the first 3 months are crucial for the restoration of consciousness.

The limitations of this study include the following:

1. The remote method for determining the level of consciousness at the post-hospital stage, which does not exclude errors in the differential diagnosis of DOC. To minimize erroneous results, we abandoned the scale assessment, which can only be carried out with direct contact with the patient.
2. The relatively short duration of the study. In the context of the tendencies towards the abolition of the term “permanent” in relation to DOC, it is more rational to assess the above indicators at a longer stage.

## 5. Conclusions

The objective of the study was to collect and assess the prognostic value of demographic, anamnestic, and clinical data based on long-term results (up to 12 months) in patients with severe acquired brain injuries in VS/UWS or MCS.

Despite the aforementioned limitations, the study showed that the demographic and clinical characteristics of patients with DOC can help predict long-term mortality. Namely, younger age and higher CRS-R score can be called predictors of a favorable outcome in terms of survival. In terms of etiology, the most unfavorable prognosis in terms of survival and consciousness change is presented in the group with ABI. ABI and, to a lesser extent, VL, were unfavorable predictors of possible increase in the level of consciousness compared to TBI.

It is important to note that the overwhelming majority of patients who have increased their level of consciousness and acquired the ability to communicate do not recover functionally and require control and a complex of rehabilitation measures from relatives and social services.

Further studies should be devoted to the peculiarities of the course of complications of the immobilization syndrome in all subgroups of the DOC, since a high level of consciousness in combination with severe functional deficit does not relieve somatic risks, which is also demonstrated in our study. No significant correlations were found between survival and improvement in the level of consciousness with the patient’s gender characteristics.

The authors consider as their further task the continuation of this longitudinal study up to 3 and 5 years after the brain lesion.

## Figures and Tables

**Table 1:** Published data on survival and consciousness improvement in DOC patients.

No.	Study	Number of Patients According to the Level of Consciousness	Number of Patients according to the Etiology	Survival Rate, Level of Consciousness/Number of Patients/Years of Follow-Up	Improvement of Consciousness, Level of Consciousness/Number of Patients/Years of Follow-Up
1	Nakase-Richardson et al., 1977 [8]	VS/UWS + MCS—396	TBI—396	VS/UWS + MCS/368/2	VS/UWS + MCS/28/3
2	Higashi et al., 1977 [9]	VS/UWS—10, MCS—0	TBI—about 1/3, VL—about 1/5	VS/UWS/65/1 VS/UWS/39/3	VS/UWS/9/1
3	Luaute et al., 2010 [10]	VS/UWS—12, MCS—39	TBI—18, ABI—16, Other—17	VS/UWS/3/5 MCS/25/5	VS/UWS/0/5 MCS/13/5
4	Estraneo et al., 2010 [11]	VS/UWS—50, MCS—0	TBI—18, ABI—18, VL—14	VS/UWS/29/1	VS/UWS/6/1
5	Estraneo et al., 2013 [12]	VS/UWS—43, MCS—0	ABI—43	VS/UWS/19/2	VS/UWS/9/2
6	Steppacher et al., 2014 [13]	VS/UWS—59, MCS—43	Not specified	VS/UWS + MCS/29/2	VS/UWS + MCS/12/2
7	Baricich et al., 2017 [14]	VS/UWS—49, MCS—0	ABI—24, TBI—12, VL—11	VS/UWS/20/4	VS/UWS/6/4
8	Aidinoff et al., 2017 [15]	VS/UWS—206, MCS—0	TBI—154, ABI—14, VL—30, Other—8	VS/UWS/139/1 VS/UWS/107/2 VS/UWS/105/3	VS/UWS/111/3
9	Faugeras et al., 2017 [16]	VS/UWS—33, MCS—34	TBI—13, ABI—19, Other—35	VS/UWS/5/0.5–3 MCS/20/0.5–3	No data available
10	Yelden et al., 2017 [17]	VS/UWS—27, MCS—7	ABI—15, TBI—6, VL—13	No data available	VS/UWS + MCS/11/2 - 16
11	Pascarella et al., 2018 [18]	VS/UWS—37, MCS—16	TBI—12, ABI—11, VL—30	VS/UWS + MCS/37/6	VS/UWS + MCS/17/6
12	Estraneo et al., 2019 [19]	VS/UWS—159, MCS—57	VL—96, ABI—71, TBI—49	VS/UWS/131/1 VS/UWS/105/2 VS/UWS/92/3 MCS/41/1 MCS/34/2 MCS/32/3	VS/UWS/35/1 VS/UWS/8/2 VS/UWS/0/3 MCS/26/1 MCS/1/2 MCS/1/3
13	Chen et al., 2020 [20]	VS/UWS—52, MCS—31	ABI—10, TBI—35, VL—48	VS/UWS + MCS/7/1	VS/UWS + MCS/33/3
14	Estraneo et al., 2020 [21]	VS/UWS—71 MCS—76	ABI—36, TBI—55, VL—56	VS/UWS/60/0.5 MCS/72/0.5	VS/UWS + MCS/72/0.5

ABI—anoxic brain injury, TBI—traumatic brain injury, VL—vascular lesions, VS/UWS—vegetative state/unresponsive wakefulness syndrome, MCS—minimally-conscious state.

**Table 2:** Demographical and clinical data of patients at study entry.

Mean (Median) Age	44.99 ± 15.69 (43 (32, 59)			
Sex (Male/Female)	122/89			
Etiology	Total (Male/Female)	VS/UWS	MCS−	MCS+
Anoxia	53 (26/27)	40	12	1
TBI	93 (65/28)	44	25	24
Vascular lesions	59 (28/31)	35	16	8
Other	6 (3/3)	4	2	0
Total level of consciousness		123	55	33
Mean CRS-R score	7.34 ± 3.67	5.081 ± 1.22	8.22 ± 2.2	14.33 ± 1.83

**Table 3:** Mortality within first year after brain injury.

Level of Consciousness	At 3 Months	At 6 Months	At 12 Months
Total: 74	15	22	37
VS/UWS	13	8	20
MCS−	1	6	11
MCS+	1	4	6
Coma		3	
Conscious		1	

**Table 4:** Changes in the level of consciousness at the 3 month checkpoint.

Level of Consciousness	Improvement (the Level of Consciousness Achieved) (out of Them Died)	Impairment (the Level of Consciousness Achieved) (out of Them Died)	No Change (out of Them Died)
Total: 211	49 (20 in MCS−; 21 in MCS+; 8 conscious) (1)	5 (2 in coma; 3 in MCS−) (1)	157 (13)
VS/UWS	27 (20 in MCS−; 5 in MCS+; 2 conscious)	0	96 (12)
MCS−	17 (16 in MCS+; 1 conscious) (1)	1 (1 in coma)	37 (1)
MCS+	5 (5 conscious)	4 (1 in coma; 3 in MCS−) (1)	24

**Table 5:** Changes in the level of consciousness at the 6 month checkpoint.

Level of Consciousness	Improvement (the Level of Consciousness Achieved) (out of Them Died)	Impairment (the Level of Consciousness Achieved) (out of Them Died)	No Change (out of Them Died)
Total: 196	32 (15 in MCS−; 12 in MCS+; 5 conscious) (3)	5 (2 in coma; 2 in MCS−; 1 in VS/UWS) (2)	159 (17)
VS/UWS	18 (15 in MCS−; 3 in MCS+) (1)	1 (1 in coma) (1)	65 (8)
MCS−	10 (9 in MCS+, 1 conscious) (2)	1 (1 in VS/UWS)	48 (5)
MCS+	4 (4 conscious)	3 (1 in coma, 2 in MCS−) (1)	37 (3)
Coma			1 (1)
Conscious			8

**Table 6:** Changes in the level of consciousness at the 12 month checkpoint.

Level of Consciousness	Improvement (the Level of Consciousness Achieved) (out of Them Died)	Impairment (the Level of Consciousness Achieved) (out of Them Died)	No Change (out of Them Died)
Total: 174	14 (2 in MCS−; 6 in MCS+; 6 conscious) (4)	1 (1 in VS/UWS) (1)	159 (32)
VS/UWS	5 (2 in MCS−; 2 in MCS+; 1 conscious) (2)		53 (19)
MCS−	6 (4 in MCS+; 2 conscious) (2)		53 (11)
MCS+	3 (3 conscious)	1 (in VS/UWS) (1)	41 (2)
Coma			
Conscious			12

**Table 7:** The number and growth in communicating, non-communicating, and deceased patients of various etiologies.

Etiology	C, init	N, init	C, 3 m	N, 3 m	D, 3 m	C, 6 m	N, 6 m	D, 6 m	C, 12 m	N, 12 m	D, 12 m
ABI	1	52	3 (+2)	43 (−9)	7	5 (+2)	36 (−7)	12 (+5)	5	24 (−12)	24 (+12)
TBI	24	69	31 (+7)	61 (−8)	1	33 (+2)	55 (−6)	5 (+4)	37 (+4)	34 (−21)	22 (+17)
VL	8	51	18 (+10)	36 (−15)	5	18	24 (−12)	17 (+12)	17 (+2)	18 (−6)	24 (+7)
Other	0	6	0	4 (−2)	2	1 (+1)	2 (−2)	3 (+1)	4 (+3)	2	4 (+1)

C—communicating, N—non-communicating, D—deceased, init—initial, m—month, +—increased, −—decreased. The deceased patients were calculated cumulatively.

**Table 8:** Logistic regression model for 211 patients.

Factor	Reference	*β*	*p*	OR (95% CI)
Age		–0.05	<0.001	0.94 (0.92–0.97)
Gender (M)	F	–0.17	0.61	0.84 (0.43–1.63)
Etiology (Traumatic)	Non-Traumatic	–0.12	0.74	0.88 (0.42–1.84)
Status (Non-Communicating)	Communicating	–0.65	0.47	0.52 (0.08–3.2)
CRS-R		0.25	<0.001	1.29 (1.08–1.53)

OR = odds ratio; CI = confidence intervals; M = male; F = female; CRS-R = coma recovery scale-revised; communicating = MCS+, non-communicating = VS/UWS and MCS−.

**Table 9:** Logistic regression model for 137 patients.

Factor	Reference	*β*	*p*	OR (95% CI)
Age		0.01	0.26	1.02 (0.99–1.04)
Gender (M)	F	0.12	0.77	1.13 (0.49–2.63)
Etiology (Traumatic)	Non-Traumatic	–0.47	0.29	0.62 (0.25–1.51)
CRS-R		–0.13	0.05	0.88 (0.77–1.00)

## Data Availability

The data are not publicly available due to the nature of this research (some information could compromise the privacy of research participants), but upon reasonable request, some data that support the findings of this study are available from the corresponding author, Pradhan P.
